# Endoscopic Ultrasound-Guided Gastrojejunostomy for Malignant Afferent Loop Syndrome Using a Fully Covered Metal Stent: A Multicenter Experience

**DOI:** 10.3390/jcm12103524

**Published:** 2023-05-17

**Authors:** Saburo Matsubara, Sho Takahashi, Naminatsu Takahara, Keito Nakagawa, Kentaro Suda, Takeshi Otsuka, Yousuke Nakai, Hiroyuki Isayama, Masashi Oka, Sumiko Nagoshi

**Affiliations:** 1 Department of Gastroenterology and Hepatology, Saitama Medical Center, Saitama Medical University, Saitama 350-8550, Japan; 2 Department of Gastroenterology, Graduate School of Medicine, Juntendo University, Tokyo 113-8431, Japan; 3 Department of Gastroenterology, Graduate School of Medicine, The University of Tokyo, Tokyo 113-8655, Japan

**Keywords:** endoscopic ultrasound, gastrojejunostomy, afferent loop syndrome

## Abstract

Background: Endoscopic-ultrasound-guided gastrojejunostomy (EUS-GJ) can be a new alternative for patients with malignant afferent loop syndrome (MALS). However, a fully covered self-expandable metal stent (FCSEMS) has not been well investigated in this setting. Methods: This is a multicenter retrospective cohort study. Consecutive patients that underwent EUS-GJ using a FCSEMS for MALS between April 2017 and November 2022 were enrolled. Primary outcomes were technical and clinical success rates. Secondary outcomes were adverse events, recurrent symptoms, and overall survival. Results: Twelve patients (median age: 67.5 years (interquartile range: 58–74.8); 50% male) were included. The most common primary disease and type of previous surgery were pancreatic cancer (67%) and pancreatoduodenectomy (75%), respectively. Technical success and clinical success were achieved in all patients. Procedure-related adverse events occurred in one patient (8%) with mild peritonitis. During a median follow-up of 96.5 days, one patient (8%) had recurrent symptoms due to the EUS-GJ stent dysfunction; including biliary events unrelated to the EUS-GJ stent, five patients (42%) had recurrent events. The median overall survival was 137 days. Nine patients (75%) died due to disease progression. Conclusions: EUS-GJ with a FCSEMS seems safe and effective for MALS with high technical and clinical success rates and an acceptable recurrence rate.

## 1. Introduction

Malignant afferent loop syndrome (MALS) is a rare late complication after surgery for upper abdominal malignancies caused by mechanical obstruction of the afferent loop after Billroth II or Roux-en-Y reconstruction due to local recurrence or peritoneal dissemination [1]. The dilated blind loop is filled with intestinal, bile, and pancreatic fluid, which can cause severe symptoms such as abdominal pain, nausea/vomiting, cholangitis, sepsis, and perforation, requiring rapid decompression [2,3]. In the past, surgery was applied but was too invasive for patients with advanced malignancies, and percutaneous drainage was problematic because of the risk of peritonitis and the loss of quality of life resulting from an external drainage [4].

Recently, minimally invasive endoscopic procedures (transmural or transluminal) have been reported [5]. Of these, transmural procedures, which anastomose digestive tracts using endoscopic ultrasound (EUS) with a double pigtail plastic stent (DPPS) [6,7], fully covered self-expandable metal stent (FCSEMS) [8,9], or lumen apposing metal stent (LAMS) [10,11,12,13,14,15], have been increasingly reported because of their easiness and high success rate.

LAMSs with an electrocautery tip (Hot LAMSs) can be placed in a single step without device exchange, which reduces the procedure time and leakage of gastrointestinal fluid. In addition, leakage of gastrointestinal fluid after stent placement is virtually prevented because the gastrointestinal tracts are firmly adhered to each other. These features are thought to be highly effective in preventing peritonitis [16]. However, LAMSs are the most costly and there are concerns about late adverse events such as bleeding [17] and buried stents [10], so the safety of long-term indwelling has not been established. Conversely, LAMS removal appears to lead to closure of the gastro-jejunal anastomosis [18]. DPPSs are the least costly, but their lack of self-expanding properties and need for several device exchanges for needle puncture, tract dilation, and stent insertion carry the risk of peritonitis due to the leakage of gastrointestinal contents, and their small diameter may shorten the stent patency [7]. FCSEMSs also require multiple device exchanges and do not have the adherence effect between the gastrointestinal tracts, so the risk of peritonitis seems higher than with LAMSs, but seems lower than with PSs because of their self-expanding properties. In addition, the diameter and cost are between those of DPPSs and LAMSs. On the other hand, FCSEMSs may be at higher risk of migration than LAMSs and DPPSs due to the lack of reliable anti-migration properties. To prevent FCSEMS migration, coaxial placement of a DPPS [8] in a FCSEMS or the use of a FCSEMS with flares [19] has been reported.

Given these facts, FCSEMSs can be suitable stents for EUS-guided MALS treatment, but only a few reports have been published using FCSEMSs. Therefore, a multicenter study was conducted to evaluate the usefulness of FCSEMSs on EUS-guided gastrojejunostomy (EUS-GJ) in patients with MALS.

## 2. Patients and Methods

### 2.1. Patients

This was a retrospective cohort study performed at three tertiary centers. The study protocol was collectively reviewed and approved by the institutional review board of Saitama Medical Center, Saitama Medical University (ethical approval number 2022-101), then permission to conduct the study was obtained at each institution. The medical records were queried for all consecutive patients who underwent EUS-GJ with a FCSEMS for relief of symptoms of MALS between April 2017 and November 2022. MALS was diagnosed when computed tomography (CT) showed obstruction and proximal dilatation of the afferent loop due to local recurrence or peritoneal dissemination of the previously resected tumor with symptoms such as abdominal pain, fever, cholangitis/jaundice, or nausea/vomiting. Considering the effect on bile excretion, the site of afferent loop obstruction was classified according to the previous report [20] as follows: Type 1: distal to the bile outlet (duodenal papilla or biliojejunal anastomosis); Type 2: including the bile outlet; Type 3: proximal to the bile outlet. The diagnosis of cholangitis was made according to Tokyo guidelines 2018 [21].

### 2.2. Procedures

**Figure 1:**
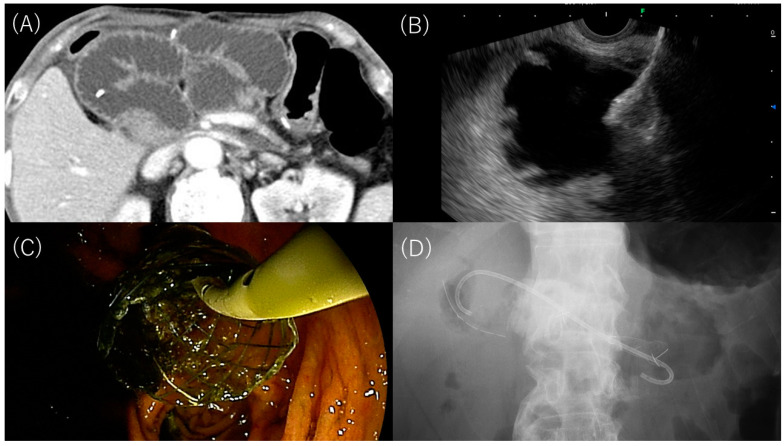
EUS-GJ for malignant afferent syndrome after pancreatoduodenectomy. (**A**) CT showed the dilated afferent loop. (**B**) Opening of the proximal end of a dumbbell-shaped FCSEMS. (**C**) A coaxial DPPS insertion through the FCSEMS. (**D**) The FCSEMS and DPPS were placed.

EUS-GJ was performed using an oblique-viewing curved-linear array echoendoscope (EG-580UT or EG-740UT; Fujifilm Medical Corp, Tokyo, Japan) and a dedicated processor (SU-1; Fujifilm Medical Corp). Patients were sedated in the prone position with midazolam and pethidine hydrochloride. The dilated afferent loop was punctured with a 19-gauge needle (EZ Shot 3 Plus; Olympus Medical Systems, Tokyo, Japan) under ultrasound guidance avoiding the intervening vessels evaluated in Doppler mode. After aspirating the intestinal fluid and confirming needle entry, a small amount of contrast medium was injected to depict the afferent loop. A 0.025-inch guidewire (EndoSelector; Boston Scientific Japan, Tokyo, Japan or VisiGlide 2; Olympus Medical Systems) was passed through the needle and coiled in the afferent loop. The needle was removed, leaving the guidewire in situ, then the tract was dilated. Dilation was performed using a bougie (ES dilator; Zeon Medical, Tokyo, Japan), balloon dilator (REN; Kaneka Medix, Osaka, Japan), or diathermic dilator (6-Fr Cystotome; Endo-Flex GmbH, Voerde, Germany or Fine025; Medico’s Hirata, Osaka, Japan). Following the tract dilation, a dumbbell-shaped FCSEMS with a 10 or 12 mm diameter (HILZO stent; BCM Ltd., Seoul, Korea) or a conventional tubular-type FCSEMS with a 10 mm diameter (WallFlex; Boston Scientific Japan) was placed between the afferent loop and stomach. The HILZO stent has dumbbell-shaped flares at both ends that are 4 mm larger in diameter than the stent body, which are expected to prevent stent migration. Therefore, this stent was preferentially used, but at one institution (Saitama Medical Center, Saitama Medical University), this stent became available in the middle of the study period, so the WallFlex was used in the beginning. The dilatation method, stent diameter, and stent length (6 or 8 cm) were selected by the endoscopist. An additional 7-Fr double pigtail plastic stent (DPPS) (Zimmon; Cook Medical, Bloomington, IN, USA or Through & Pass; Gadelius Medical, Tokyo, Japan) was coaxially placed through the metal stent to prevent migration at the discretion of the endoscopist (Figure 1).

All patients underwent plain CT and laboratory tests on the next day of the procedure. An oral diet was started when clinical symptoms improved without any severe adverse event.

### 2.3. Definitions and Outcome Measures

Primary outcomes were technical and clinical success. Technical success was defined as the appropriate placement of a FCSEMS as intended. Clinical success was defined as relief of MALS-related symptoms and decompression of the dilated afferent loop on CT findings within one week. Secondary outcomes were early (up to 14 days) and late adverse events, recurrent symptoms, and overall survival (OS). Adverse events were described in accordance with the American Society for GI Endoscopy lexicon [22].

### 2.4. Statistical Analyses

Descriptive continuous variables were presented as number (percentage) or median (interquartile range (IQR)). The Kaplan–Meier method was used to estimate recurrent symptom-free survival (RFS) and OS. Deaths without recurrent symptoms were treated as censored at the time of death in RFS. The follow-up data were gathered until March 2023. All statistical analyses were performed with EZR Ver. 1.52 (Saitama Medical Center, Jichi Medical University, Saitama, Japan) [23], which is a graphical user interface for R (The R Foundation for Statistical Computing, Vienna, Austria).

## 3. Results

### 3.1. Patient Characteristics

Twelve patients (median age: 67.5 years (IQR: 58–74.8); 50% male) were enrolled in this study. Baseline characteristics are shown in Table 1. The most common primary disease was pancreatic cancer (67%). The types of previous surgery were pancreatoduodenectomy (75%) and hepatectomy with hepaticojejunostomy (25%). Obstruction types were 42%, 42%, and 17% for Types 1, 2, and 3, respectively. The major symptoms of MALS were fever (67%) and abdominal pain (58%), while cholangitis or obstructive jaundice were present in 50% of patients. A small amount of ascites was seen in 58% of cases on CT, but no ascites was seen between the stomach and afferent loop. EUS observation during the EUS-GJ procedure also showed no ascites.

Prior afferent loop drainage had been attempted in three cases (25%) (transluminal approach in two cases and percutaneous approach in one case). The transluminal approach was performed using a double-balloon endoscope (EI-580BT; Fujifilm Medical Corp), with one case unsuccessful because the guidewire could not pass through the stricture, and in the other case, a nasal tube was placed. The percutaneous approach was performed transhepatically with the placement of an external tube. The nasal and percutaneous tubes were removed after conversion to EUS-GJ later. Prior biliary drainage had been performed in four cases (endoscopic retrograde cholangiopancreatography (ERCP) using a double-balloon endoscope in two cases, EUS-guided hepaticogastrostomy (EUS-HGS) in one case, and the combination of both in one case), all of which were Type 2 cases.

### 3.2. EUS-Guided Gastrojejunostomy

Technical success was achieved in 12 cases (100%). Details and outcomes of procedures are shown in Table 2. The diathermic dilator, bougie plus balloon dilator, and balloon dilator were used to dilate the tract in 58%, 33%, and 8% of cases, respectively. In the FCSEMS used, 83% were dumbbell-shaped, 58% were 10 mm in diameter, and 75% were 8 cm in length. The coaxial DPPS was placed in all but one case with a dumbbell-shaped FCSEMS. The median procedure time was 31 min (IQR: 19.3–42.5 min). Clinical success was achieved in 12 cases (100%). There was only one early adverse event of mild peritonitis (8%), which was resolved conservatively with antibiotics.

### 3.3. Follow-Up

**Figure 2:**
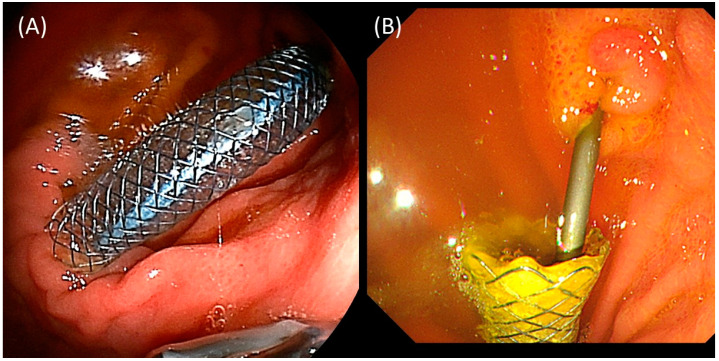
Migration of a tubular-type FCSEMS. (**A**) A tubular-type SEMS and coaxial DPPS were successfully indwelled. (**B**) Seventy-two days later, the FCSEMS was completely migrated outward, but the DPPS kept the anastomosis.

Long-term outcomes after EUS-GJ procedures are shown in Table 3. During the follow-up period (median: 96.5 days (IQR: 45.3–210.8)), no late adverse events other than the recurrence of MALS-related symptoms occurred. The recurrence of MALS-related symptoms developed in five cases (42%) with six episodes of biliary events. One biliary event was due to EUS-GJ stent dysfunction: the patient with a Type 1 obstruction indwelling a tubular-type FCSEMS and coaxial DPPS had recurrent jaundice due to outward migration of the FCSEMS. In this case, the DPPS was still in situ and the anastomosis was not closed. Therefore, the FCSEMS and DPPS were removed, and a guidewire was inserted through the anastomosis using an ERCP catheter. Finally, two new DPPSs were placed and the jaundice was improved (Figure 2). The remaining five events were newly developed obstructive jaundice/cholangitis due to tumor progression in three cases and recurrent biliary obstruction due to the dysfunction of existing biliary stents in two cases. Newly developed obstructive jaundice/cholangitis included Type 1 in two cases and Type 3 in one case. EUS-HGS was performed in two cases (one case each of Type 1 and Type 3). In one case of Type 1, EUS-HGS could not be performed, because of post-left-hepatectomy; ERCP using an ultra-slim endoscope via the EUS-GJ anastomosis was attempted but failed; and PTBD was finally performed. Two cases of recurrent biliary obstruction due to the dysfunction of existing biliary stents were both Type 2 cases (one each after ERCP and EUS-HGS) and underwent endoscopic stent exchange.

**Figure 3:**
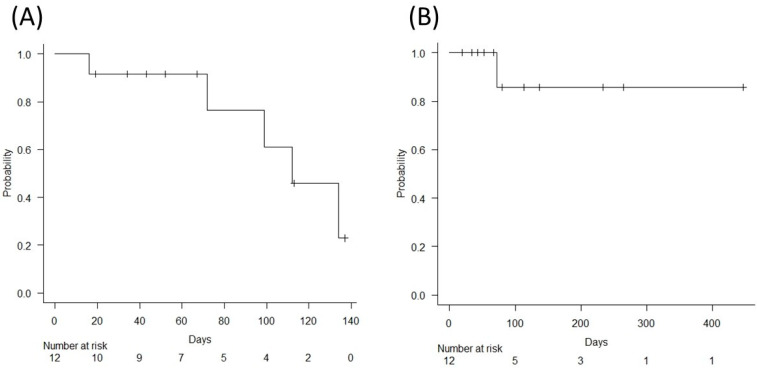
Recurrent symptom-free survival (RFS). (**A**) Including all events. Median RFS (95% CI) was 112 days (72-NA), and the recurrence-free probability at 90 days (95% CI) was 0.764 (0.309–0.940). (**B**) Including only events due to EUS-GJ stent dysfunction. Median RFS (95% CI) and recurrence-free probability at 90 days (95% CI) were NA (72-NA) and 0.857 (0.334–0.979), respectively.

The median RFS (95% confidence interval (CI)) was 112 days (72-not applicable (NA)), and the recurrence-free probability at 90 days (95% CI) was 0.764 (0.309–0.940). When recurrent events were limited to those due to EUS-GJ-stent dysfunction, recurrence occurred in one case (8%), with a median RFS (95% CI) of NA (72-NA) and recurrence-free probability at 90 days (95% CI) of 0.857 (0.334–0.979) (Figure 3). Median OS (95% CI) was 137 days (43–265). Nine patients (75%) died due to progression of the underlying disease.

## 4. Discussion

In the present study, both technical and clinical success rates of EUS-GJ with a FCSEMS for MALS were excellent at 100%, with an acceptable adverse events rate of 8%. Recurrence of symptoms developed in 42%, but most were biliary events unrelated to the EUS-GJ-stent; when recurrent events were limited to those due to EUS-GJ-stent dysfunction, symptom recurrence was only 8%, and the recurrence-free probability at 90 days was 0.857. To the best of our knowledge, this study represents the largest series of patients that underwent EUS-GJ with a FCSEMS for MALS.

Currently, minimally invasive endoscopic procedures are the mainstay for MALS. There are two types of endoscopic procedures: transluminal and EUS-guided transmural approaches [2,5]. The transluminal approach has become easier with the advent of balloon-assisted endoscopes with a large-diameter channel and duodenal SEMSs with a slim introducer that allow through-the-scope placement of a SEMS [20,24]. However, it is still difficult to reach the stricture site in cases of severe postoperative adhesions or peritoneal dissemination [7]. In addition, stent dysfunction rates were considerably high (0–38%) due to tumor ingrowth in uncovered SEMSs and migration in covered SEMSs [25]. Gutierrez et al. reported an indirect comparison of transmural and transluminal approaches, showing a significantly lower reintervention rate for the former (16.6% vs. 76.5%, *p* < 0.001) [12]. Similarly, a comparison of EUS-GJ and duodenal stenting for malignant gastric outlet obstruction reported a lower reintervention rate with EUS-GJ (1% vs. 26%, *p* < 0.001) [26]. Furthermore, it may be more difficult in the transluminal approach for reintervention to reach the stricture site due to further tumor progression. Although prospective comparative studies are lacking, EUS-GJ would be the first choice for MALS considering the easiness of the procedure, low reintervention rate, and reliability of the reintervention procedure.

There are two reports of EUS-GJ with a LAMS for MALS involving more than 10 cases: Gutierrez et al. reported a technical success rate of 100%, clinical success rate of 89%, adverse events rate of 17%, and reintervention rate of 17% during a median follow-up of 120 days in 18 patients [12]; Perez-Cuadrado-Robles et al. reported a technical success rate of 96%, clinical success rate of 91%, adverse events rate of 4.4%, and reintervention rate of 15% during a median follow-up of 4 months in 45 patients [10]. The present study was comparable to these reports in terms of success and adverse events rates, and the reintervention rate was also comparable when recurrent events were limited to EUS-GJ-stent dysfunction.

The advantage of the LAMS is its large diameter. However, as the contents of the afferent loop are liquid, a large diameter is not necessary. In fact, there was no difference in efficacy according to the diameter of the LAMS [10]. Nevertheless, in cases where EUS-HGS is not possible for obstructive jaundice, as in the case of PTBD in this study, the possibility of ERCP via the anastomosis can be an advantage of the large-diameter LAMS [10].

Another advantage of the LAMS (especially Hot LAMS) is that the one-step placement and the adherent effect of the gastrointestinal tracts minimize leakage of gastrointestinal fluids, preventing peritonitis. The FCSEMS requires multiple device exchanges to dilate the tract, increasing the risk of peritonitis. Although a 6-Fr cystotome is the most reliably insertable dilation device, a balloon was used by endoscopists who were concerned about bleeding due to the cystotome in this study. The balloon has the risk of pushing on the afferent loop and dislodging the guidewire, so a bougie dilatation followed by balloon dilatation was often used. As a result, peritonitis developed in one of the 12 cases (8%). In this case, the insertion of a dumbbell-type FCSEMS was attempted after dilatation with a 6-Fr cystotome but failed. The stent could be inserted by changing the guidewire from 0.025 inches to 0.035 inches with the use of an ERCP catheter. Because the FCSEMS introducer is as thick as 8.5-Fr, it could not fit in the 6-Fr cystotome dilation. In addition, the patient had a high fever immediately before the procedure and was considered to have had an active infection in the afferent loop. Multiple device exchanges in the presence of an active infection caused peritonitis. The optimal dilation method for FCSEMS placement is unclear and remains an issue for future study.

Migration is the major drawback of the FCSEMS and was observed in 1/12 (8%) of the patients in the present study. Fortunately, the coaxial DPPS could maintain the anastomosis, which facilitates reintervention. Migration was observed in 1/2 (50%) of the tubular type, but 0/10 (0%) of the dumbbell-shaped type, suggesting that the anti-migration function of the dumbbell-shaped FCSEMS was effective. A DPPS should be added when using a tubular-type FCSEMS. LAMSs are short in length and have large flanges at both ends that firmly adhere the digestive tract to each other, making migration unlikely. On the other hand, “buried LAMS” can occur, in which the stent is buried within the gastrointestinal wall.

Obstructive jaundice and cholangitis are common manifestations in Type 1 and 2 MALS. In Type 1, EUS-GJ alone is sufficient because the biliojejunal anastomosis is spared, whereas biliary drainage is usually required in Type 2. Biliary drainage may be necessary even in Type 1 and 3 patients with disease progression [10], as in the three cases (EUS-HGS in two cases and PTBD in one case) in the present study. When fever develops after EUS-GJ, the possibility of cholangitis as well as EUS-GJ stent obstruction should be taken into consideration.

The present study has several limitations. First, this was a retrospective study with a limited number of patients, so selection bias and reporting bias could not be eliminated. Second, the patient characteristics such as the primary disease and surgical method, as well as the procedure details such as the dilation method, the type and size of FCSEMS used, and the combination with a DPPS, were disparate and not standardized. Third, it was a single-arm study with no comparison arm. The strengths of this study are its largest patient population in this setting and its multicenter design.

In conclusion, EUS-GJ with a FCSEMS seems safe and effective for MALS with high technical and clinical success rates and an acceptable recurrence rate. Further investigations including comparative studies with EUS-GJ with a LAMS are warranted in the near future.

## Figures and Tables

**Table 1:** Patient characteristics.

Age, years	67.5 (58–74.8)
Sex, male	6 (50)
ECOG-PS, 1/2/3	8 (67)/3 (25)/1 (8)
Primary disease	
Pancreatic cancer	8 (67)
Cholangiocarcinoma	3 (25)
Gallbladder cancer	1 (8)
Type of previous surgery	
Pancreatoduodenectomy	9 (75)
Hepatectomy with hepaticojejunostomy	3 (25)
Cause of obstruction	
Local recurrence	7 (58)
Peritoneal dissemination	5 (42)
Type of obstruction, 1/2/3	5 (42)/5 (42)/2 (17)
Time from surgery to drainage, days	523 (312–1019)
Symptoms of MALS	
Fever	8 (67)
Abdominal pain	7 (58)
Cholangitis/Jaundice	6 (50)
Nausea/vomiting	2 (17)
Presence of ascites	7 (58)
Prior afferent loop drainage	2 (17)
Transluminal naso-tube drainage	1 (8)
Percutaneous transhepatic drainage	1 (8)
Prior biliary drainage	4 (33)
ERCP	2 (17)
EUS-HGS	1 (8)
Combination	1 (8)
C-reactive protein, mg/dL	2.35 (0.75–5.9)
Total bilirubin, mg/dL	2.0 (0.75–4.85)
Alkaline phosphatase, U/L	610.5 (309–846.3)

Numbers are shown in number (%) or median (interquartile range). ECOG-PS, Eastern Cooperative Oncology Group performance status; MALS, malignant afferent loop syndrome; ERCP, endoscopic transpapillary cholangiopancreatography; EUS-HGS, endoscopic-ultrasound-guided hepaticogastrostomy.

**Table 2:** Details and outcomes of endoscopic-ultrasound-guided gastrojejunostomy procedures.

Technical success	12 (100)
Clinical success	12 (100)
Dilation method	
Diathermic	7 (58)
Bougie plus Balloon	4 (33)
Balloon	1 (8)
Fully covered self-expandable metal stent	
Type, Dumbbell-shaped/Tubular	10 (83)/2 (17)
Diameter, 10 mm/12 mm	7 (58)/5 (42)
Length, 8 cm/6 cm	9 (75)/3 (25)
Use of a coaxial double pigtail plastic stent	11 (92)
Procedure time, min	31 (19.3–42.5)
Early adverse events	1 (8)
Mild peritonitis	1 (8)

Numbers are shown in number (%) or median (interquartile range).

**Table 3:** Long-term outcomes after procedures.

Follow-up period, days	96.5 (45.3–210.8)
Late adverse events other than recurrence of MALS-related symptoms	0 (0)
Recurrence of MALS-related symptoms, patients/episodes	5 (42)/6
Recurrent jaundice due to EUS-GJ-stent dysfunction (migration)	1 (8)
Newly developed obstructive jaundice/cholangitis due to tumor progression	3 (25)
Recurrent biliary obstruction due to existing biliary stents’ dysfunction	2 (17)
Reintervention, patients/sessions	5 (42)/6
EUS-GJ-stent exchange	1 (8)
EUS-HGS	2 (17)
PTBD	1 (8)
ERCP-stent exchange	1 (8)
EUS-HGS-stent exchange	1 (8)
Median overall survival, days (95% CI)	137 (43–265)

Numbers are shown in number (%) or median (interquartile range). EUS-GJ, endoscopic-ultrasound-guided gastrojejunostomy; CI, confidence interval; EUS-HGS, endoscopic-ultrasound-guided hepaticogastrostomy; ERCP, endoscopic retrograde cholangiopancreatography; PTBD, percutaneous transhepatic biliary drainage.

## Data Availability

The data generated during this study are available within the article. Datasets analyzed during the current study preparation are available from the corresponding author on reasonable request.
